# Association of Complement Factor D and H Polymorphisms with Recurrent Pregnancy Loss

**DOI:** 10.3390/ijms21010017

**Published:** 2019-12-18

**Authors:** Hee Young Cho, Han Sung Park, Eun Ju Ko, Chang Soo Ryu, Jung Oh Kim, Young Ran Kim, Eun Hee Ahn, Woo Sik Lee, Nam Keun Kim

**Affiliations:** 1 Department of Obstetrics and Gynecology, CHA Bundang Medical Center, CHA University, Seongnam 13496, Korea; hycho.md@gmail.com (H.Y.C.); happyiran@cha.ac.kr (Y.R.K.); bestob@chamc.co.kr (E.H.A.); 2 Department of Biomedical Science, College of Life Science, CHA University, Seongnam 13488, Korea; hahnsung@naver.com (H.S.P.); ejko05@naver.com (E.J.K.); regis2040@nate.com (C.S.R.); jokim8505@gmail.com (J.O.K.); 3 Fertility Center of CHA Gangnam Medical Center, CHA University, Seoul 06125, Korea

**Keywords:** complement factor D, complement factor H, recurrent pregnancy loss, polymorphism

## Abstract

Recurrent pregnancy loss (RPL) is defined as two or more consecutive pregnancy losses prior to 20 weeks of gestation, and the incidence of RPL is estimated at 1% of all pregnancies. While the etiologies of RPL are diverse, immune function is considered to be an important cause of RPL. In particular, the complement system is essential for stable development of the placenta and fetus. Moreover, complement factor D (*CFD)* and complement factor H (*CFH)* are important regulators of the complement system and are associated with diseases, such as age-related macular degeneration. Therefore, we investigated whether polymorphisms of *CFD* and *CFH* are associated with RPL in 412 women with RPL and 384 control women. Genotyping of three polymorphisms (*CFD* rs2230216, *CFH* rs1065489, and *CFH* rs1061170) was performed by TaqMan probe real-time PCR and PCR-restriction fragment length polymorphism. Association of three polymorphisms with RPL was evaluated by statistical analysis. The GT/TC genotype combination of *CFH* rs1065489 G>T/*CFH* rs1061170 T>C was associated with a decreased risk of RPL occurrence compared with reference genotypes (adjusted odds ratio [AOR] = 0.439; 95% confidence interval [CI] = 0.238–0.810; *p* = 0.008), and this association remained significant after adjustment for multiple comparisons using false discovery rate (FDR) correction (*p* = 0.040). In addition, the *CFH* rs1065489G>T polymorphism is associated with homocysteine and prolactin level and *CFH* rs1061170 TC genotype is related to uric acid and triglycerides level in RPL patients. Therefore, those factors could be possible clinical risk factors in RPL patients.

## 1. Introduction

Recurrent pregnancy loss (RPL) is defined as two or more consecutive pregnancy losses prior to 20 weeks of gestation [1,2]. Approximately 10–12% of all pregnant women experience early pregnancy loss within 8–12 weeks of pregnancy [3], and the incidence of RPL is estimated at 1% of all pregnancies [4]. RPL is an important clinical issue in reproductive health but, unfortunately, the etiological factors cannot be identified in more than half of RPL patients [5]. In cases with identifiable causes, genetic disorders, such as fetal chromosomal abnormalities; maternal factors, including anatomical deformities, placental anomalies, thrombophilia, endocrine disorders, immune dysfunction, infection, smoking, psychological trauma, and stress; and environmental factors have been implicated in RPL [6,7]. Among these factors, immune function is considered an important cause of RPL, as pregnancy induces a complex immune response at the implantation site to facilitate and protect the pregnancy and prevent response to the “foreign” fetus [8].

The complement system is a pivotal facet of the innate immune system, reinforcing the function of antibodies and promoting phagocytic cells to remove infectious agents and apoptotic cells. In addition, the complement system connects the innate immune response and the acquired immune system via activated complement fragments [9]. Previous studies revealed that improper complement activation, either too little or too much, leads to poor pregnancy outcomes. Specifically, exaggeration of complement activation in the placenta results in placental damage and increased risk of preeclampsia and fetal loss [10,11].

Among the complement cascade players, C1q is the primary factor involved in the regulation of fetal survival through trophoblast migration and spiral artery remodeling. C1q-deficient mice exhibit abnormal placental development and subsequent altered fetal size and weight [12]. In addition, increased anti-C1q antibodies provoke a placental defect that is a consequence of the failure of trophoblast migration, spiral artery remodeling, and normal placentation, implicating this response in miscarriage in unexplained RPL [13]. Moreover, C3 is essential for the activation of the classical and alternative pathways of the complement system. This factor also plays an important role in early placental development, and several C3 gene variants have been found in association with idiopathic RPL [14,15,16]. In animals, C3 in rats on the visceral yolk sac plays an important embryotrophic factor in early post-implantation rat embryo, and adding intact C3 in explant rat embryo culture significantly facilitates embryo development. Moreover, C3 knock-out mice showed smaller blastocysts and placentas and higher resorption rates compared to normal mice [17].

C3b activation is regulated by factor I in the presence of complement factor H (*CFH)*, which is a cofactor for factor I. Factor I is involved in C3 cleavage and has decay-accelerating activity against C3 convertase (C3Bb), thus inhibiting C3b cleavage and acting as a regulator to appropriately regulate complement activity [18]. Interestingly, Tan et al. [19] recently reported that genetic variants of *CFH* may be associated with the histopathologic subtypes and clinical features in Chinese lupus nephritis patients. Complement factor D (*CFD)* cleaves factor B, which is a constituent of the complement activation pathway, into a non-catalytic unit Ba and a catalytic unit Bb, and the active Bb acts as a serine protease that together with complement C3b, forms the C3-converting enzyme [20]. C3 protein is regulated by both *CFH* and *CFD* and C3 has been found to be associated with RPL. Therefore, we hypothesized that *CFH* and *CFD* genetic variants are associated with RPL through the regulation of *C3* (Figure S1). In this study, we evaluated the relationship between *CFH* and *CFD* polymorphisms and susceptibility to RPL.

## 2. Results

The baseline characteristics and laboratory test values of the women in the RPL and control groups were evaluated (Table 1). There were no significant differences in age or body mass index (BMI) between the two groups. Women with RPL had significantly higher hematocrit (Hct), platelets (PLT), activated partial thromboplastin time (aPTT), blood urea nitrogen (BUN), creatinine, luteinizing hormone (LH), and estradiol (E_2_) and lower prothrombin time (PT), total cholesterol, and follicle-stimulating hormone (FSH) than women in the control group. Analysis of the genotype frequencies of *CFD* and *CFH* in RPL patients and controls (Table 2) revealed that the *CFH* rs1061170 T>C polymorphism was significantly associated with RPL risk (adjusted odds ratio [AOR] = 0.625; 95% confidence interval [CI] = 0.409–0.954; *p* = 0.029), although this association did not remain significant after adjustment using the false discovery rate [FDR] correction (*p* = 0.116).

Different combinations of the *CFD* and *CFH* genotypes may affect the modifiers of RPL risk. Therefore, the combinations of the *CFD* and *CFH* genotypes were investigated for associations with risk of RPL (Table 3). The genotype combination of CG/TT for *CFD* rs2230216C>G/*CFH* rs1065489G>T exhibited a significant association with increased risk of RPL, but the difference was no longer significant after FDR correction (*p* = 0.108). On the other hand, the GT/TC genotype for *CFH* rs1065489G>T/*CFH* rs1061170T>C was associated with a decreased risk of RPL compared with reference genotypes, and this association remained significant after FDR adjustment (*p* = 0.040). 

We evaluated whether the allele combinations have synergistic effects on RPL risk (Table S1). The GC combination of *CFH* rs1065489 G>T/*CFH* rs1061170 T>C was associated with a decreased risk of RPL occurrence compared with reference genotypes (AOR = 0.510; 95% CI= 0.311–0.837; *p* = 0.007), and this association remained significant after adjustment for multiple comparisons using FDR correction (*p* = 0.021).

**Figure 1:**
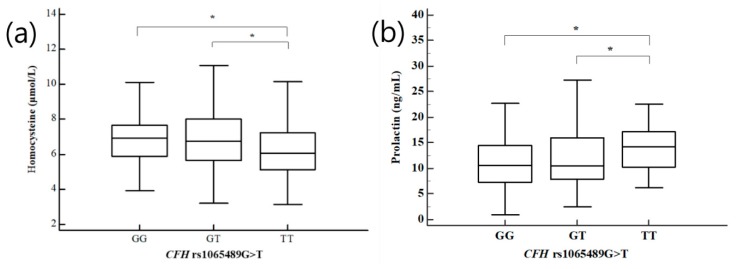
Association between (**a**) homocysteine and (**b**) prolactin levels and the *CFH* rs1065489G>T polymorphisms in patients with RPL. * *p* < 0.05.

**Figure 2:**
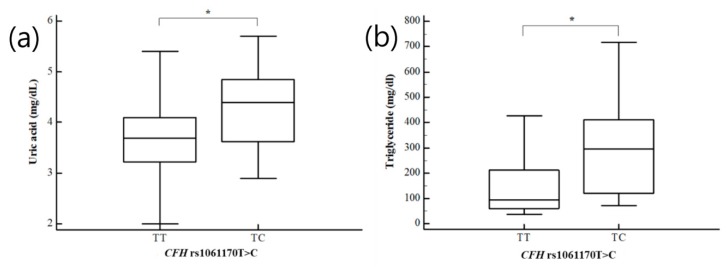
Association between (**a**) uric acid and (**b**) triglyceride levels and the *CFH* rs1061170T>C polymorphisms in patients with RPL. * *p* < 0.05.

As previous research indicated clinical parameters that were different in women with RPL, we examined the combined effects of *CFD* and *CFH* genotypes and clinical risk factors on the odds of RPL (Table S2). The *CFH* rs1061170 TC genotype and BMI < 25 kg/m^2^ were associated with a decreased RPL risk (AOR = 0.551, 95% CI: 0.352–0.861, *p* = 0.027 after FDR correction). Differences in plasma levels of clinical risk-associated factors in recurrent pregnancy loss patients, such as uric acid, homocysteine, FSH, prolactin, and triglycerides, were evaluated for the three *CFD* and *CFH* genotypes (Table S3). Significant differences in uric acid, homocysteine, and prolactin were identified for the GG, GT, and TT genotypes of *CFH* rs1065489G>T. The TT and TC genotypes of the *CFH* rs1061170 T>C exhibited statistically significant differences in plasma levels of uric acid, FSH, and triglycerides. We compared homocysteine and prolactin levels in women with RPL according to the GG, GT, and TT genotypes of *CFH* rs1065489G>T (Figure 1). Women with RPL and *CFH* rs1065489TT genotype had significantly lower homocysteine levels than women with RPL and *CFH* rs1065489 GG and GT genotypes. In addition, higher prolactin levels were found in patients with the *CFH* rs1065489TT genotype compared to patients with the *CFH* rs1065489GG and GT genotypes. Patients with the *CFH* rs1061170TC genotype had significantly higher uric acid and triglyceride levels than patients with the *CFH* rs1061170TT genotype (Figure 2). However, ANOVA analysis of the control group did not show statistically significant results. We showed the association between homocysteine levels and the *CFH* rs1065489G>T polymorphism in control group (Figure S2).

## 3. Discussion

In the present study, we investigated the correlation between *CFD* and *CFH* polymorphisms and risk of RPL. We analyzed the effects of diverse alleles and genotypes of *CFD* rs2230216, *CFH* rs1065489, and *CFH* rs1061170. The GT/TC combination for *CFH* rs1065489G>T/*CFH* rs1061170T>C was significantly associated with a lower risk of RPL.

CFH has an important role in the alternative complement activation pathway, regulating C3 activation and protecting the tissues from inflammatory injury. Previous studies probed the relationship between CFH and systemic lupus erythematosus susceptibility [21,22]. CFH prevents the formation of C3 convertase and also facilitates the decomposition of C3 convertase and the degradation of C3b. CFH contains 20 short consensus repeats (SCRs). SCR1-4 in the N-terminus mediate the cofactor/decomposition-facilitating activity, and SCR19-20 in the C-terminus are essential for cell surface regulation of CFH. In recent years, multiple *CFH* SNPs have been investigated for association with human diseases, such as age-related macular degeneration (AMD). The most studied *CFH* SNP is rs1061170, which is located in SCR7 and regulates the binding of CFH to C-reactive protein (CRP) and glycosaminoglycans (GAGs) [23,24,25]. Moreover, the genotype of *CFH* rs1061170 was found to be related to specific histopathologic subtypes of lupus nephritis [16], highlighting the relevance of complement polymorphisms in inflammatory disease.

Those three polymorphisms such as *CFD* rs2230216 C>G, *CFH* rs1065489 G>T, and *CFH* rs1061170 C>T are missense variants located on the coding region of each gene. The alteration of *CFD* rs2230216 C>G, *CFH* rs1065489 G>T, and *CFH* rs1061170 C>T lead to alteration of amino acid p.Ile248Met, p.Glu936Asp, and p.His402Tyr, respectively. Those alterations of amino acids may cause changing of amino acid characteristics and effect on functions of those proteins. The *CFH* rs1065489 polymorphism was reported that associated with plasma levels of CFH. Moreover, minor genotype (TT) of *CFH* rs1061170 led to downregulation of plasma CFH levels and effect on other related complement cascade proteins including complement factor B (CFB) in transgenic mice [26,27].

In this study, *CFH* rs1061170T>C itself was not significantly associated with RPL risk after adjustment using FDR correction, but the combination of GT/TC for *CFH* rs1065489G>T/*CFH* rs1061170T>C was associated with reduced risk of RPL. Previous studies showed that *CFH* rs10611170 was present significantly more often in AMD patients than in controls. These studies also suggested that the CC/CT genotype-coding variant of rs10611170 led to decreased binding to the CRP and GAG chains, resulting in an excessive local inflammatory reaction and inhibition of complement turnover in AMD [28,29]. Similarly, reduced binding of CFH to GAGs and CRP was found to be related to immune damage in SLE and lupus nephritis [16].

One potential explanation of the protective relationship between this *CFH* polymorphism and RPL risk involves secondary genetic factors and clinical factors. Alternatively, multiple interaction sites for C3b, GAGs, or heparin with CFH have been reported, and CFH is a known ligand for CRP, adrenomodulin, and osteopontin [30]. Thus, as these numerous interactions and functions may play a role in RPL, it is difficult to pinpoint the exact mechanism underlying the relationship between CFH polymorphisms and RPL.

Furthermore, effects of *CFD* polymorphisms on RPL have not been reported previously. We did not find a significant relationship between *CFD* polymorphism and susceptibility to RPL. Among three complement activation pathways, the alternative pathway initiates activation of a C3 convertase by CFD and CFB, leading to generation of C3a and C3b. Then, C3b is converted to C3bBb by CFD and CFB and activates C5 to produce the C5b-9 membrane attack complex, causing cell lysis. Based on this pathway, we hypothesize that CFD increases susceptibility to RPL via control of C3 and C5 function indirectly; however, additional research is necessary to delineate the functions of *CFD* polymorphism in RPL. Moreover, we acknowledge that RPL results from complex pathologic causes, requiring a combined analysis of both *CFH* genes and other predisposing factors in order to understand this health problem. In our study, the levels of prolactin and triglyceride were not investigated in controls. The maintenance of the correlation of polymorphism and risk factor in the control group is important because that may become evidence about the genotype of polymorphism’s direct effect on the risk factor. We thought that the correlation might be still important and the specific correlation in patient group is more interesting. However, a study about this correlation in the control group is needed and we considered that the absence of the study is a limitation of this study.

In conclusion, this study analyzed the relationship between *CFH* and *CFD* polymorphisms and RPL. Our results indicate that *CFH* polymorphisms are significantly associated with decreased risk of RPL and support additional investigations to define the mechanism underlying this relationship.

## 4. Materials and Methods

### 4.1. Subjects

Women who presented at the Infertility Medical Center of CHA Bundang Medical Center between March 1999 and February 2012 were enrolled in the study. Written informed consent, which included information about this study, was obtained from all participants. The study was approved by the institutional review board at the CHA Bundang Medical Center (IRB number: 2010-01-123). The study group included 384 women in the control group and 412 women with RPL. We recruited women who had experienced two or more consecutive spontaneous abortions confirmed by human chorionic gonadotropin levels, sonography, and physical examination for inclusion in the RPL group. Participants who had a history of smoking or alcohol use were excluded from this study. Age-matched healthy women were selected for the control group. These women had one or more pregnancies resulting in childbirth without pregnancy complications and no history of pregnancy loss or infertility. We collected the blood samples in nonpregnant women at the time of enrollment. Baseline blood tests were performed to examine typical miscarriage causes, such as thyroid diseases and hyperprolactinemia.

### 4.2. Genotyping

Genomic DNA samples were extracted from anticoagulated peripheral blood using the G-DEX blood extraction kit (Intron, Seongnam, Korea). Genotyping of *CFD* polymorphisms was performed using the TaqMan SNP Genotyping Assay Kit (Applied Biosystems, Foster City, CA, USA) with a real-time PCR machine (RG-6000; Corbett Research, Mortlake, Australia) according to the manufacturer’s instructions. PCR-restriction fragment length polymorphism analysis was performed to genotype two polymorphisms of complement factor H. The primer pairs for *CFH* rs1065489 G>T (forward: 5′- GGC ATT GTT TAC CAG GCA TAG -3′ and reverse: 5′- AAT TGC AGG CCC ATC AAT TCC -3′) and *CFH* rs1061170 T>C (forward: 5′- GGG CCA AGA AAA GAG TTG TTC AAG C -3′, and reverse: 5′- AGG ATG GCA GGC AAC GTC TAT AGA T -3′) were designed to amplify the region of each polymorphism. The following conditions were used for PCR amplification of each polymorphism: initial denaturation at 95 °C for 15 min; 35 cycles of denaturation at 95 °C for 30 s, annealing for 30 s, and extension at 72 °C for 30 s; and final extension at 72 °C for 5 min. The annealing temperatures for the PCR amplification of *CFH* rs1065489 G>T and *CFH* rs1061170 T>C were 59 °C and 63 °C, respectively, according to the melting temperature of each primer pair. Restriction enzyme digestions were performed using *Dde*I for *CFH* rs1065489 G>T and *Nla*III for *CFH* rs1061170 T>C at 37 °C for 16 h, and inactivation of the enzyme was achieved by incubation at 65 °C for 20 min.

### 4.3. Assessment of Clinical Risk Factors

Clinical risk factor information, such as age and BMI, was recorded for each subject. We obtained peripheral blood from all subjects. We evaluated plasma levels of uric acid, cholesterol, high-density lipoprotein (HDL) cholesterol, triglyceride, folic acid, BUN, creatinine, and homocysteine in recurrent pregnancy loss patients. In addition, we obtained data for PLT counts, aPTT, PT, and CD56+ natural killer (NK) cells from peripheral blood. Uric acid, total cholesterol, HDL cholesterol, and triglycerides were measured using commercially available enzymatic colorimetric tests (Roche Diagnostics, Mannheim, Germany). Folic acid, BUN, and creatinine were measured by competitive immunoassay using an ACS:180 (Bayer Diagnostics, Whippany, NJ, USA). Fluorescence polarization immunoassay (Abbott Laboratories, Lake Bluff, IL, USA) was used to determine the levels of homocysteine. PLT counts, PT, and aPTT were measured to assess blood coagulation. PLT counts were measured using a Sysmex XE2100 automated hematology analyzer (Sysmex, Kobe, Japan). PT and aPTT were measured using an automated photo-optical coagulometer (ACL TOP; Mitsubishi Chemical Medicine, Tokyo, Japan).

### 4.4. Data Analysis

For statistical analysis, the two-sample *t*-test or the Mann–Whitney test was used for continuous variables. The association between genotypes and RPL development was determined by *p*-values, AORs, and 95% CIs. Allele frequencies were investigated for deviation from the Hardy–Weinberg equilibrium (HWE). The allele combinations for the polymorphisms were estimated with a chi-square test and were adjusted using a FDR correction. A *p* ≤ 0.05 was considered statistically significant. ANOVA and the Kruskal–Wallis test were used to examine the different clinical parameters in RPL women according to the genotypes of the three polymorphisms in *CFD* and *CFH*. The relationships between genotypes and clinical risk factors were evaluated using multiple regression analysis. Analyses were performed using Medcalc (v. 12.7.1.0, Medcalc Software, Mariakerke, Belgium) and GraphPad Prism (v. 4.0, GraphPad Software Inc. San Diego, CA, USA). The HAPSTAT program (v. 3.0, www.bios.unc.edu/~lin/hapstat/) was used for synergistic effect of the allele combination.

## Figures and Tables

**Table 1:** Baseline characteristics between patients with recurrent pregnancy loss (RPL) and controls.

Characteristic	Controls (*n* = 384)	RPL Patients (*n* = 412)	*p ^a^*
Age (years, mean ± SD)	32.84 ± 4.17	33.09 ± 4.30	0.4224
BMI (kg/m^2^)	21.78 ± 3.29	21.48 ± 3.87	0.459 ^b^
Live birth (*n*, mean ± SD)	1.64 ± 0.57	-	
Pregnancy loss (*n*, mean ± SD)	-	3.27 ± 1.83	
Gestational age at the termination of previous pregnancy (week, mean ± SD)	39.21 ± 1.66	7.41 ± 1.89	
IVF treatment (*n*, pregnancy loss *n* (mean ± SD)			
- Nontreatment	-	334 (3.38 ± 1.98)	
- One cycle	-	33 (2.65 ± 1.29)	
- Two cycles	-	41 (3.18 ± 1.22)	
- Three cycles	-	3 (3.50 ± 1.29)	
Hematocrit (μmol/L)	35.76 ± 4.10	37.25 ± 3.69	0.0001
PLT (10^3^/µL)	237.61 ± 61.07	255.37 ± 59.05	0.003
PT (sec)	11.52 ± 3.36	11.32 ± 1.76	0.0001 ^b^
aPTT (sec)	29.92 ± 4.24	32.02 ± 4.25	0.0001
BUN (mg/dL)	8.03 ± 2.01	9.95 ± 2.69	<0.0001 ^b^
Creatinine (mg/dL)	0.69 ± 0.08	0.73 ± 0.13	0.025 ^b^
Uric acid (mg/dL)	4.19 ± 1.44	3.80 ± 0.82	0.340 ^b^
Total cholesterol (mg/dl)	239.00 ± 85.19	187.70 ± 49.06	0.004 ^b^
Folate (nmol/L)	13.71 ± 8.37	16.94 ± 19.70	0.887 ^b^
Homocysteine (μmol/L)	7.28 ± 1.58	6.91 ± 2.06	0.536
FSH (mIU/mL)	8.12 ± 2.85	7.76 ± 11.47	<0.0001 ^b^
LH (mIU/mL)	3.26 ± 1.76	6.37 ± 11.95	<0.0001 ^b^
E2 (pg/mL)	26.00 ± 14.75	43.55 ± 72.70	0.0002 ^b^
TSH (µIU/mL)	-	2.16 ± 1.52	-
Prolactin (ng/mL)	-	15.35 ± 12.76	-
Triglyceride (mg/dL)	-	181.42 ± 156.63	-
HDL cholesterol (mg/dL)	-	61.82 ± 17.63	-
FBS (mg/dL)	-	95.05 ± 16.87	-

^a^ Two-sided *t*-test. ^b^ Mann–Whitney test. BMI, body mass index; IVF, in-vitro fertilization; PLT, platelet; PT, prothrombin time; aPTT, activated partial thromboplastin time; BMI, body mass index; BUN, blood urea nitrogen; FSH, follicle-stimulating hormone; LH, luteinizing hormone; E2, estradiol; TSH, thyroid-stimulating hormone; HDL cholesterol, high-density lipoprotein cholesterol; FBS, fasting blood sugar; SD, standard deviation.

**Table 2:** Genotype frequencies of complement factor D (*CFD*) and complement factor H (*CFH*) between patients with RPL and controls.

Genotypes	Controls (*n* = 384)	RPL Patients (*n* = 412)	AOR (95% CI) *	*p*	FDR-*p*
***CFD* rs2230216 C>G**					
CC	306 (79.7)	317 (76.9)	1.000 (reference)		
CG	72 (18.8)	93 (22.6)	1.225 (0.866–1.732)	0.252	0.397
GG	6 (1.6)	2 (0.5)	0.302 (0.060–1.513)	0.145	0.305
Dominant (CC vs. CG+GG)			1.154 (0.822–1.621)	0.408	0.643
Recessive (CC+CG vs. GG)			0.296 (0.059–1.481)	0.138	0.290
HWE-*p*	0.461	0.078			
***CFH* rs1065489 G>T**					
GG	109 (28.4)	123 (29.9)	1.000 (reference)		
GT	199 (51.8)	208 (50.5)	0.921 (0.666–1.272)	0.617	0.648
TT	76 (19.8)	81 (19.7)	0.931 (0.620–1.398)	0.731	0.768
Dominant (GG vs. GT+TT)			0.926 (0.682–1.258)	0.624	0.655
Recessive (GG+GT vs. TT)			0.983 (0.693–1.396)	0.925	0.971
HWE-*p*	0.387	0.680			
***CFH* rs1061170 T>C**					
TT	325 (84.6)	370 (89.8)	1.000 (reference)		
TC	59 (15.4)	42 (10.2)	0.625 (0.409–0.954)	0.029	0.091
CC	0 (0.0)	0 (0.0)	N/A	N/A	N/A
Dominant (TT vs. TC+CC)			0.625 (0.409–0.954)	0.029	0.091
Recessive (TT+TC vs. CC)			N/A	N/A	N/A
HWE-*p*	0.103	0.276			

* The odds ratio was adjusted by age. *CFD*, complement factor D; *CFH*, complement factor H; RPL, recurrent pregnancy loss; AOR, adjusted odds ratio; 95% CI, 95% confidence interval; HWE, Hardy–Weinberg equilibrium; FDR, false-positive discovery rate.

**Table 3:** Gene combination for the *CFD* and *CFH* polymorphisms in patients with RPL and controls.

Genotype combination	Controls (*n* = 384)	RPL Patients (*n* = 412)	AOR (95% CI) *	*p*	FDR-*p*
***CFD* rs2230216C>G/*CFH* rs1065489G>T**					
CC/GG	86 (22.4)	100 (24.3)			
CC/GT	151 (39.3)	157 (38.1)	0.897 (0.623–1.000)	0.561	0.813
CC/TT	69 (18.0)	60 (14.6)	0.747 (0.476–1.173)	0.205	0.646
CG/GG	23 (6.0)	23 (5.6)	0.859 (0.450–1.640)	0.645	0.813
CG/GT	44 (11.5)	49 (11.9)	0.951 (0.575–1.571)	0.843	0.885
CG/TT	5 (1.3)	21 (5.1)	3.443 (1.239–9.569)	0.018	0.113
GG/GT	4 (1.0)	2 (0.5)	0.421 (0.075–2.369)	0.326	0.685
GG/TT	2 (0.5)	0 (0.0)	N./A	N./A	N./A
***CFD* rs2230216C>G/*CFH* rs1061170T>C**					
CC/TT	260 (67.7)	283 (68.7)	1.000 (reference)		
CC/TC	46 (12.0)	34 (8.3)	0.673 (0.419–1.082)	0.102	0.293
CG/TT	60 (15.6)	85 (20.6)	1.277 (0.880–1.000)	0.199	0.293
CG/TC	12 (3.1)	8 (1.9)	0.607 (0.244–1.511)	0.283	0.297
GG/TT	5 (1.3)	2 (0.5)	0.347 (0.066–1.811)	0.209	0.293
GG/TC	1 (0.3)	0 (0.0)	N./A	N./A	N./A
***CFH* rs1065489G>T/*CFH* rs1061170T>C**					
GG/TT	92 (16.4)	108 (32.6)	1.000 (reference)		
GG/TC	17 (3.0)	15 (4.5)	0.752 (0.356–1.588)	0.454	0.563
GT/TT	161 (28.6)	188 (56.8)	0.991 (0.699–1.404)	0.957	0.804
GT/TC	38 (6.8)	20 (6.0)	0.439 (0.238–0.810)	0.008	0.034
TT/TT	72 (18.8)	74 (18.0)	0.863 (0.563–1.325)	0.501	0.563
TT/TC	4 (1.0)	7 (1.7)	1.489 (0.422–5.246)	0.536	0.563

* The odds ratio was adjusted by age. *CFD*, complement factor D; *CFH*, complement factor H; RPL, recurrent pregnancy loss; OR, odds ratio; 95% CI, 95% confidence interval; FDR, false discovery rate.

## Supplementary Materials

Supplementary materials can be found at https://www.mdpi.com/1422-0067/21/1/17/s1.
