# Supplementary material for: Association of Complement Factor D and H Polymorphisms with Recurrent Pregnancy Loss
Source: Int J Mol Sci. 2019 Dec 18;21(1):17. doi: 10.3390/ijms21010017 (PMC6981708; doi:10.3390/ijms21010017)
Supplement: Supplementary file 1 [file ijms-21-00017-s001.pdf]

Supplementary Table 1. Allele combination for the *CFD* and *CFH* polymorphisms in patients with RPL and controls.

**Allele combination**	**Controls (2n=768)**	**RPL patients (2n=824)**	**OR (95% CI)**	***p*** [a]	**FDR-*p***
***CFD* rs2230216C>G/*CFH* rs1065489G>T/*CFH* rs1061170T>C**					
C-G-T	322 (42.0)	384 (46.6)	1.000 (reference)		
C-G-C	41 (5.3)	26 (3.2)	0.532 (0.318 - 0.889)	0.015	0.053
C-T-T	310 (40.4)	303 (36.8)	0.820 (0.660 - 1.018)	0.072	0.149
C-T-C	11 (1.4)	13 (1.6)	0.991 (0.438 - 2.243)	0.983	1.000
G-G-T	49 (6.3)	44 (5.3)	0.753 (0.488 - 1.161)	0.198	0.277
G-G-C	6 (0.7)	0 (0.0)	0.065 (0.004 - 1.150)	0.009[b]	0.053
G-T-T	28 (3.6)	51 (6.2)	1.527 (0.941 - 2.479)	0.085	0.149
G-T-C	2 (0.3)	3 (0.3)	1.258 (0.209 - 7.577)	1.000[b]	1.000
***CFD* rs2230216C>G/*CFH* rs1065489G>T**					
C-G	363 (47.3)	410 (49.7)	1.000 (reference)		
C-T	321 (41.8)	317 (38.5)	0.874 (0.709 - 1.079)	0.210	0.210
G-G	54 (7.0)	44 (5.4)	0.721 (0.473 - 1.101)	0.129	0.194
G-T	30 (3.9)	53 (6.4)	1.564 (0.978 - 2.502)	0.060	0.180
***CFD* rs2230216C>G/*CFH* rs1061170T>C**					
C-T	633 (82.4)	688 (83.5)	1.000 (reference)		
C-C	51 (6.7)	39 (4.8)	0.704 (0.457 - 1.082)	0.108	0.170
G-T	76 (10.0)	94 (11.5)	1.138 (0.825 - 1.569)	0.430	0.452
G-C	8 (1.0)	3 (0.3)	0.345 (0.091 - 1.307)	0.101[b]	0.170
***CFH* rs1065489G>T/*CFH* rs1061170T>C**					
G-T	371 (48.3)	427 (51.8)	1.000 (reference)		
G-C	46 (6.0)	27 (3.3)	0.510 (0.311 - 0.837)	0.007	0.018
T-T	338 (44.0)	355 (43.1)	0.913 (0.744 - 1.119)	0.379	0.398
T-C	13 (1.7)	15 (1.8)	1.003 (0.471 - 2.135)	0.995	0.697

[a] Chi-square test. [b] Fisher's exact test. CFD, complement factor D; CFH, complement factor H; RPL, recurrent pregnancy loss; OR, odds ratio; 95% CI, 95% confidence interval; FDR, false discovery rate.

Supplementary Table 2. Synergistic effects of complement genotypes and clinical factors in RPL risk.

Variables	CFD rs2230216 CC					CFD rs2230216 CG + GG				
	Control	RPL	AOR (95% CI)	*p*	FDR-*p*	Control	RPL	AOR (95% CI)	*p*	FDR-*p*
**PLT**										
$< 304\times10^3$ /μl	149	130	1.000 (reference)			29	42	1.154 (0.812 - 1.642)	0.425	0.446
$\geq 304\times10^3$/μl	20	32	1.600 (0.893 - 2.865)	0.114	0.359	4	8	1.939 (0.575 - 6.540)	0.286	0.446
**PT**										
> 10.4 sec	28	163	1.000 (reference)			4	43	1.140 (0.805 - 1.614)	0.46	0.483
≤ 10.4 sec	13	22	1.709 (0.844 - 3.462)	0.137	0.272	2	6	3.065 (0.613 - 15.334)	0.173	0.272
**aPTT**										
> 26.8 sec	55	168	1.000 (reference)			9	41	1.144 (0.808 - 1.000)	0.448	0.47
≤ 26.8 sec	22	18	1.861 (0.903 - 3.836)	0.092	0.271	3	7	3.075 (0.615 - 15.382)	0.172	0.271
**BMI**										
<25 kg/㎡	85	260	1.000 (reference)			15	79	1.185 (0.832 - 1.688)	0.346	0.242
≥25 kg/㎡	13	30	2.317 (1.183 - 4.537)	0.014	0.029	5	9	1.816 (0.601 - 5.494)	0.291	0.242

Variables	CFH rs1065489 GG					CFH rs1065489 GT +TT				
	Control	RPL	AOR (95% CI)	*p*	FDR-*p*	Control	RPL	AOR (95% CI)	*p*	FDR-*p*
**PLT**										
$< 304\times10^3$ /μl	41	47	1.000 (reference)			137	125	0.888 (0.645 - 1.222)	0.465	0.732
$\geq 304\times10^3$/μl	8	10	1.118 (0.425 - 2.943)	0.821	0.862	16	30	1.677 (0.863 - 3.258)	0.127	0.4
**PT**										
> 10.4 sec	6	58	1.000 (reference)			26	148	0.935 (0.682 - 1.283)	0.678	0.712
≤ 10.4 sec	5	10	1.840 (0.609 - 5.562)	0.28	0.441	10	18	1.687 (0.742 - 3.834)	0.212	0.441
**aPTT**										
> 26.8 sec	13	60	1.000 (reference)			51	149	0.944 (0.689 - 1.294)	0.722	0.758
≤ 26.8 sec	10	8	2.322 (0.707 - 7.632)	0.165	0.321	15	17	1.702 (0.749 - 3.868)	0.204	0.321
**BMI**										
<25 kg/㎡	32	103	1.000 (reference)			68	236	0.944 (0.686 - 1.299)	0.723	0.759
≥25 kg/㎡	6	14	2.216 (0.819 - 5.995)	0.117	0.184	12	25	1.953 (0.932 - 4.096)	0.076	0.184

Variables	CFH rs1061170 TT					CFH rs1		
	Control	RPL	AOR (95% CI)	*p*	FDR-*p*	Control	RPL	AOR (
**PLT**								
$< 304\times10^3$ /μl	29	158	1.000 (reference)			27	14	0.596 (0.
$\geq 304\times10^3$/μl	20	34	1.552 (0.874 - 2.756)	0.133	0.209	4	6	1.311 (0.
**PT**								
> 10.4 sec	29	187	1.000 (reference)			3	19	0.631 (0.
≤ 10.4 sec	12	25	1.918 (0.947 - 3.886)	0.071	0.119	3	3	0.928 (0.
**aPTT**								
> 26.8 sec	56	190	1.000 (reference)			8	19	0.633 (0.
≤ 26.8 sec	21	22	2.101 (1.016 - 4.345)	0.045	0.071	4	3	0.931 (0.
**BMI**								
<25 kg/㎡	83	306	1.000 (reference)			17	33	0.551 (0.
≥25 kg/㎡	17	32	1.705 (0.928 - 3.135)	0.086	0.06	1	7	6.376 (0.7

[*] The odds ratio was adjusted by age. RPL, recurrent pregnancy loss; *CFD*, complement factor D; *CFH*, complement factor H; AOR, adjusted odds ratio; 95% CI, 95% confidence interval; PLT, platelet; PT, prothrombin time; aPTT, activated partial thromboplastin time; BMI, body mass index.
[†] PLT 304×10^3/μl indicates the upper 15% cut-off level in RPL patients and controls.
[‡] PT 10.4 sec, aPTT 26.8 sec indicates the lower 15% cut-off levels in RPL patients and controls.

Supplementary Table 3. Clinical variables in recurrent pregnancy loss patients stratified by *CFD* and *CFH* polymorphisms status by ANOVA.

Genotypes	Uric acid (mg/dL)	Homocysteine (μmol/L)	FSH (mIU/mL)	Prolactin (ng/mL)	Triglyceride (mg/dl)
	Mean ± SD	Mean ± SD	Mean ± SD	Mean ± SD	Mean ± SD
***CFD* rs2230216 C>G**					
CC	3.87 ± 0.85	6.96 ± 2.17	8.35 ± 13.00	15.02 ± 12.84	190.08 ± 152.18
CG	3.60 ± 0.74	6.73 ± 1.65	6.14 ± 4.52	16.45 ± 12.66	152.20 ± 169.00
GG	3.65 ± 0.64	6.23 ± 2.25	2.13 ± 0.00	9.94 ± 0.00	324.00 ± 0.00
P^a	0.153	0.636	0.434	0.705	0.438
***CFH* rs1065489 G>T**					
GG	3.57 ± 0.54	7.33 ± 2.35	7.95 ± 9.89	12.20 ± 7.26	160.84 ± 157.32
GT	4.00 ± 0.91	6.95 ± 1.97	7.72 ± 8.88	15.72 ± 13.29	187.88 ± 159.03
TT	3.61 ± 0.78	6.19 ± 1.59	7.53 ± 18.26	20.44 ± 17.38	190.19 ± 158.33
P^a	0.007 [b]	0.003	0.983	0.002 [b]	0.804
***CFH* rs1061170 T>C**					
TT	3.74 ± 0.80	6.97 ± 2.11	6.74 ± 6.70	15.65 ± 13.35	165.91 ± 142.07
TC	4.28 ± 0.91	6.33 ± 1.43	16.25 ± 28.44	12.97 ± 6.10	305.50 ± 217.69
CC	-	-	-	-	-
P^a	0.010	0.102	0.035 [b]	0.324	0.016

ANOVA, analysis of variance; SD, standard deviation; FSH, follicle stimulating hormone; CFD, complement factor D; CFH, complement factor H.

[a] ANOVA test, [b] Kruskal-Wallis test.

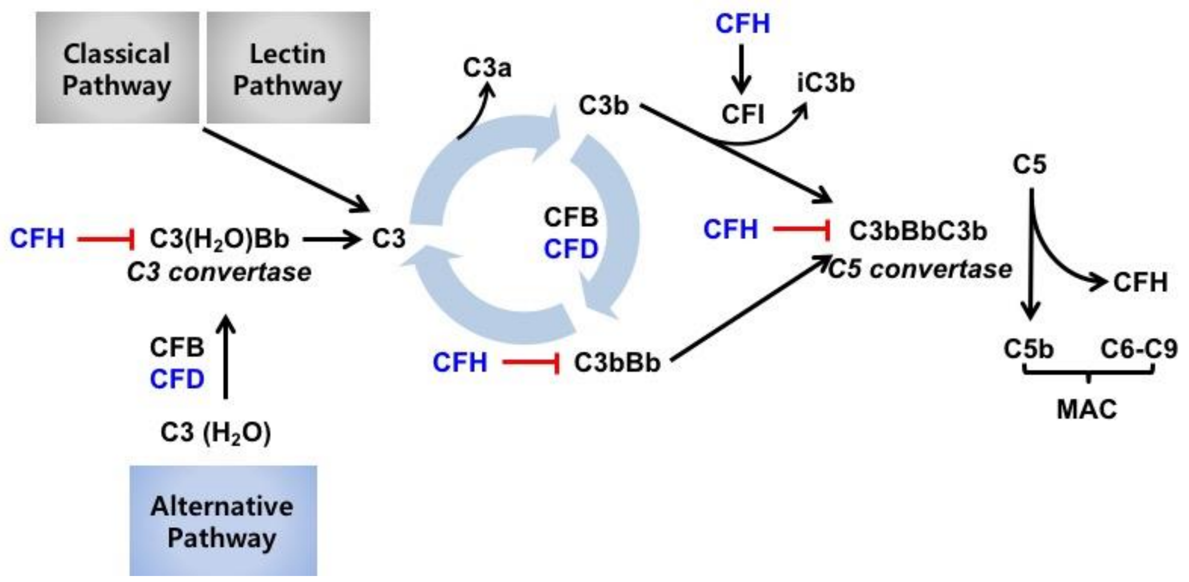

Supplementary Figure 1. The three pathways of complement activation. Black arrow indicates triggering of complement cascades. Deposition of C3b on a target sets in motion the powerful amplification loop of the alternative pathway. The C3b deposition and C3a release generate the downstream mediators C5b-9. C3 is regulated by both CFH and CFD and C3 has been found to be associated with RPL.

CFH, complement factor H; CFI, complement factor I; CFB, complement factor B; CFD, complement factor D; MAC, membrane attack complex.

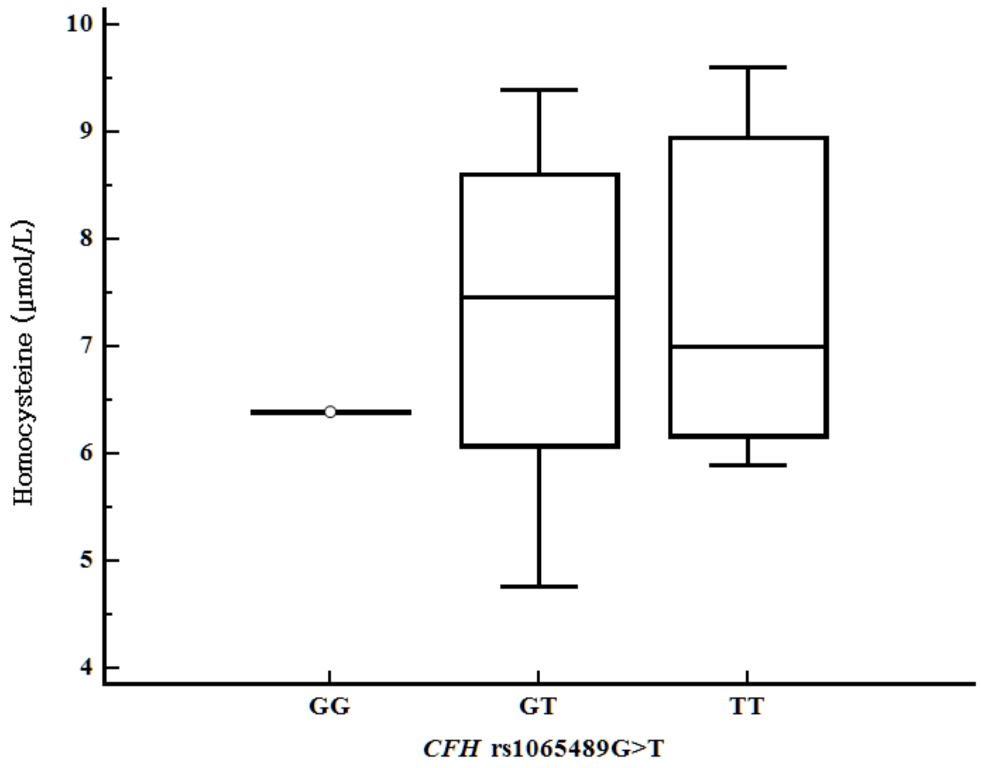

Supplementary Figure 2. Association between homocysteine levels and the *CFH* rs1065489G>T polymorphisms in control group.
